# Integrated Genomic and Phenotypic Characterization of an *Mcr-10.1*-Harboring Multidrug Resistant *Escherichia coli* Strain From Migratory Birds in China

**DOI:** 10.1155/tbed/7631217

**Published:** 2025-05-01

**Authors:** Ronglei Huang, Xue Ji, Bing Liang, Bowen Jiang, Danhong Wang, Yi Tang, Chengyang Zhang, Ang Zhou, Nan Li, Chongtao Du, Yang Sun

**Affiliations:** ^1^State Key Laboratory for Diagnosis and Treatment of Severe Zoonotic Infectious Diseases, Key Laboratory for Zoonosis Research of the Ministry of Education, Institute of Zoonosis, and College of Veterinary Medicine, Jilin University, Changchun 130062, China; ^2^Changchun Veterinary Research Institute, Chinese Academy of Agricultural Sciences, Changchun, China; ^3^Key Laboratory of Jilin Province for Zoonosis Prevention and Control, Changchun, China

## Abstract

**Background:** The global rise in antibiotic resistance among multidrug resistant (MDR) Gram-negative (GN) bacteria has posed significant health challenges, leading to the resurgence of colistin as a key defense against these bacteria. However, the widespread use of colistin has resulted in the rapid emergence of colistin resistance on a global scale. Ten members of the (mobile colistin resistance) *mcr* gene family, *mcr-1* through *mcr-10*, have been reported and documented. Currently, bacteria reported to carry the *mcr-10.1* gene are sensitive to colistin, but the mechanism underlying the low-level resistance phenomenon mediated by *mcr-10.1* remains unclear.

**Methods:** In this study, antimicrobial susceptibility testing (AST) was conducted on *Escherichia coli* (E.coli) isolated from Chinese migratory birds, resulting in the selection of 87 strains exhibiting MDR phenotypes. Whole-genome sequencing (draft) was performed on these 87 MDR *E. coli* strains, and for one of the *E. coli* strains carrying the *mcr-10.1* gene, whole-genome sequencing, phenotypic characterization, AST and conjugation experiments were conducted to identify its resistance phenotypes and genetic characteristics.

**Results:** Whole-genome sequencing (draft) of 87 MDR *E. coli* isolates revealed a diverse array of resistance genes, predominantly including aminoglycoside, *β*-lactam, tetracycline, and sulfonamide resistance genes. Remarkably, one isolate, despite being sensitive to colistin, harbored the *mcr-10.1* gene. Further sequencing showed that *mcr-10.1* was located in the conserved region of *xerC-mcr-10.1*, a hotspot for movable elements with various insertion sequences (ISs) or transposons nearby. Phenotypic characterization indicated that the MDR plasmid pGN25-*mcr10.1* had no significant effect on the growth of GN25 and its derivatives but reduced the number of bacterial flagella.

**Conclusions:** It is particularly important to note that bacteria harboring the *mcr-10.1* gene may exhibit low minimum inhibitory concentration (MIC) values, but that the MIC values under colistin selective pressure can become progressively higher and exacerbate the difficulty of treating infections caused by *mcr-10.1*-associated bacteria. Therefore, vigilance for such “silent transmission” is warranted, and continuous monitoring of the spread of *mcr-10.1* is necessary in the future.

## 1. Introduction

The plasmid-mediated mobile colistin resistance (*mcr*) gene *mcr-1* was first identified in *Enterobacteriaceae* in China [1], highlighting a critical horizontal transfer mechanism for resistance to colistin, a last-resort antibiotic in veterinary and human medicine. Subsequently, the *mcr-1* gene has demonstrated rapid evolutionary adaptation and widespread dissemination across multiple niches, spanning human populations, livestock, wildlife, fresh produce, and environmental samples from agricultural and aquatic ecosystems. In 2016, Ruzauskas and Vaskeviciute [2] detected the *mcr-1* resistance gene in *Escherichia coli* (*E. coli*) in European herring gulls *Larus argentatus*, the first time that the *mcr* resistance gene had been found in a wild bird. In 2020, a new *mcr* gene, *mcr*-10, was identified in the IncFIA plasmid of a clinical strain of *Enterobacter cloacae loganicum* [3]. SinceThe then, bacteria harboring the colistin resistance gene have been increasingly identified in wild birds in many countries, with *E. coli* predominating and with *Salmonella*, *Klebsiella pneumoniae*, and *Pseudomonas aeruginosa* detected at significantly lower prevalence rates. The horizontal transfer of mobile genetic elements (MGEs), particularly plasmids carrying *mcr* genes (with *IncX4*, *IncI2* as predominant types, followed by *IncHI2* and *IncP*), has significantly accelerated the global dissemination of colistin resistance. Epidemiological surveys confirm that these plasmid types are actively circulating across human, animal, and environmental compartments [4], although birds have not been found to carry the *mcr-10* gene so far.

Colistin is one of the last available antibiotic options for the treatment of infection with Multidrug Resistant Gram-Negative Bacilli (MDR-GNB) strains, but the extensive use of colistin has exacerbated the acquisition and spread of the *mcr* genes. To date, the *mcr* gene has been reported from several locations around the world [5]. In this study, we performed whole-genome sequencing of *E. coli* GN25, a strain harboring the *mcr-10.1* gene isolated from migratory birds, and characterized the genetic structure of the *mcr-10.1* gene. Furthermore, we constructed an isogenic plasmid-cured strain and subjected both the wild-type and plasmid-cured strains to in vitro colistin selection pressure. This comparative approach aimed to determine whether colistin exposure modulates key fitness traits in *mcr-10.1*-harboring *E. coli*.

## 2. Materials and Methods

### 2.1. Identification and Antimicrobial Susceptibility Testing (AST)

Our laboratory focuses on monitoring pathogens associated with migratory birds. In a prior study, we isolated 87 strains of MDR *E. coli* strains from migratory bird fecal samples in China [6, 7]. For this work, we used the BD Phoenix−100 system (Becton, Dickinson and Company, USA) to confirm species identification and antibiotic susceptibility profiles of these 87 *E. coli* isolates along with GN25-derived strains. We determined antimicrobial MIC values through CLSI-standardized broth microdilution assays, using *E. coli* ATCC 25922 for quality control [8, 9].

### 2.2. Whole Genome Sequencing and Analysis

The whole genome (draft) of 87 *E. coli* strains were sequenced using Illumina NovaSeq PE150 platform at the Beijing Novogene Bioinformatics Technology Co. Ltd. (Beijing, China). To ensure the accuracy and reliability of the subsequent information analysis results, the original data were filtered to obtain valid data (clean data). Subsequently, SOAP denovo software was utilized to perform genome assembly on this clean data. The acquired antimicrobial resistance genes were identified by online resfinder 4.4.0 (https://cge.cbs.dtu.dk/services/ResFinder/). Single nucleotide polymorphism (SNP)-based phylogenetic analysis was conducted by aligning core genomes using Snippy v 4.6.0, followed by maximum-likelihood tree reconstruction using RAxML v 8.2.12. The resulting phylogeny was visualized and annotated with antimicrobial resistance profiles using Interactive Tree of Life (iTOL) v 6.0 [10].

The complete closed genome assembly of *E. coli* strain GN25 was sequenced by the Beijing Novogene Bioinformatics Technology Co. Ltd. (Beijing, China) using a NovaSeq 6000 System (Illumina Inc., San Diego, CA, USA) and a PacBio Sequel platform (PacBio, Menlo Park, CA, USA). The acquired antimicrobial resistance genes were identified by online resfinder 4.4.0 (https://cge.cbs.dtu.dk/services/ResFinder/) and the Comprehensive Antibiotic Research Database (CARD) database (https://card.mcmaster.ca/). Insertion sequences (ISs) were annotated through sequence alignment against the ISfinder database. (https://isfinder.biotoul.fr/blast.php/). The plasmid types (https://cge.cbs.dtu.dk/services/PlasmidFinder/) and MLST typing (https://cge.cbs.dtu.dk/services/MLST/) was performed using the CGE website (https://cge.cbs.dtu.dk/services/). Subsequently, the resistance genes, plasmid replicons and ISs were identified and visualized using BRIG and Easyfig 2.2.3 [11]. oriTfinder online tool (https://tool-mml.sjtu.edu.cn/oriTfinder/oriTfinder.html) was used to inspect for the origins of transfer (oriTs) and other conjugation gene modules.

### 2.3. Conjugation Experiments

We performed conjugation assays using *E. coli* GN25 (donor; tetracycline-resistant) and rifampicin-resistant *E. coli* C600 (recipient). Both strains were grown overnight at 37°C in 3 mL LB broth containing either 4 mg/L tetracycline (donor) or 800 mg/L rifampicin (recipient). After mixing donor and recipient cultures (1:1 ratio), 0.1 mL aliquots were filtered through 0.22 μm nitrocellulose membranes. These membranes were then placed on LB agar and incubated for 12 h at 37°C to allow mating. To recover transconjugants, we plated the mixtures on LB agar plates supplemented with 800 mg/L rifampicin and 4 mg/L tetracycline, selecting for transfer of the *mcr-10.1*-bearing plasmid.

### 2.4. In Vitro Induction Assay and Biological Characterization of Strain GN25

The pGN25-*mcr10.1* plasmid-cured strain GN25-Q was obtained by successive passaging, and then GN25 and GN25-Q were continuously induced in vitro with colistin to produce drug-resistant phenotypes in these two strains. Following colistin induction, the derived strains were designated as GN25-Y (parental strain: GN25) and GN25-QY (parental strain: GN25-Q). Comprehensive phenotyping of all four strains included: (i) antimicrobial susceptibility profiling using the BD Phoenix−100 automated microbiology system; (ii) conjugative transfer assessment via filter mating experiments; (iii) growth kinetics monitoring in LB medium at 37°C; (iv) motility evaluation through semi-solid agar cultures; (v) cellular ultrastructure analysis by transmission electron microscopy (TEM). These investigations aimed to determine whether the presence of the plasmid-borne *mcr-10.1* gene and colistin exposure jointly modulate bacterial fitness-associated traits. The details of methodological approaches are described in the Supporting Information 1: Supporting methods.

### 2.5. Nucleotide Sequence Accession Numbers

The raw sequencing reads (Illumina NovaSeq PE150) for the 87 MDR *E. coli* strains, including the representative strain GN25, have been deposited in the NCBI Sequence Read Archive (SRA) under BioProject PRJNA1029772. The completely assembled plasmid sequences from strain GN25 are available in GenBank under accession CP139196-CP139197.

## 3. Results

### 3.1. Whole-Genome (draft) Resistance Data for MDR *E. coli*

**Figure 1:**
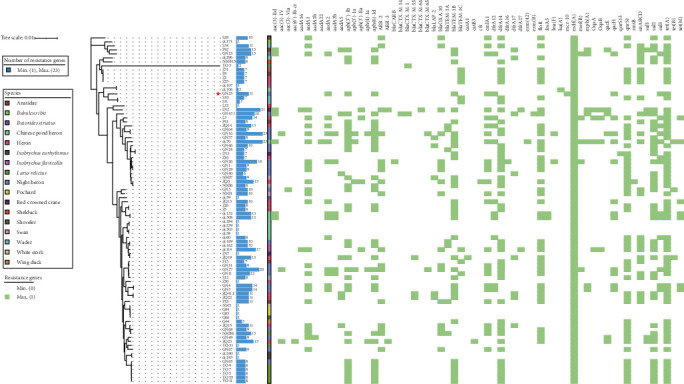
Cluster and heat map analyses. Cluster and heat map analyses showing the resistance data for *E. coli* isolates obtained from different geographic regions and the second-generation sequencing data, suggest that migratory birds have some influence on bacterial resistance transmission. The bar graphs indicate the number of resistance genes carried by each strain of *E. coli* and the colored blocks indicate the origin of the species carrying *E. coli*.

Utilizing the whole-genome (draft) sequencing data, we conducted a comprehensive analysis of the antimicrobial resistance profiles and subsequently generated a resistance heatmap for visualization (Figure 1). The results revealed that 77.01% (67/87) of the *E. coli* isolates examined harbored more than five resistance genes, with two isolates carrying a remarkable maximum of 23 resistance genes. The predominant resistance determinants encompassed aminoglycoside resistance genes (i.e., *aadA1*, *aph* (*3′'*)*-Ib*, and *aph* (*6*)*-Id*), *β*-lactam resistance genes (i.e., *bla*_CTX-M_ and *bla*_TEM_), tetracycline resistance genes (i.e., *tetA*), and sulfonamide resistance genes (i.e., *sul2* and *sul3*). This result is generally consistent with the previous PCR-based identification of resistance genes conducted in our laboratory [7]. Surprisingly, during our analysis of the whole-genome (draft) sequencing data, we discovered a strain of *E. coli*, designated as GN25, which harbors the *mcr-10* gene but remarkably exhibits no colistin resistance phenotype. (Supporting Information 2: Table S1)

### 3.2. Integrated Genomic and Comparative Plasmid Analysis of *E. coli* GN25

**Figure 2:**
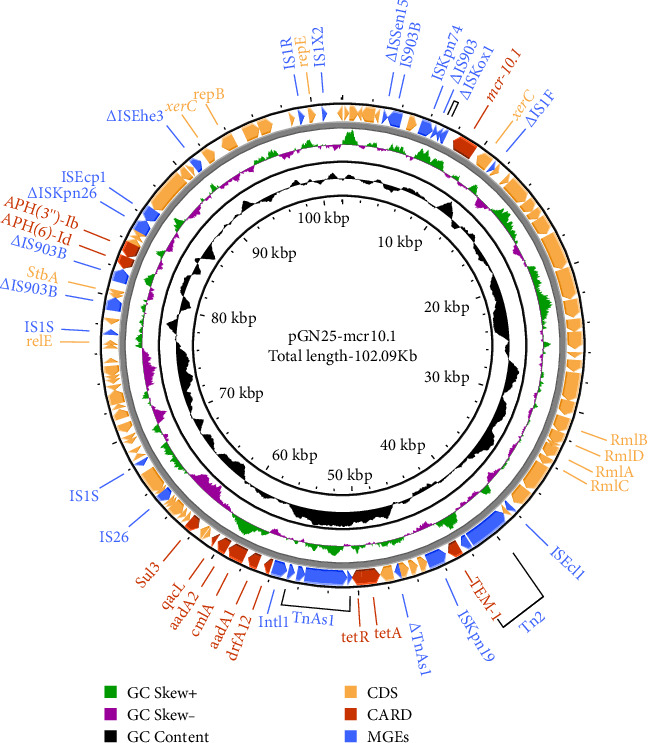
Structural analysis of plasmid pGN25-*mcr10.1*. Schematic diagram of plasmid pGN25-*mcr10.1*. The gene backbone is indicated by arrows, with blue arrows in the outer circle indicating mobile elements such as various insertion sequences and red arrows indicating drug resistance genes. The innermost ring indicates the GC content, and the ring immediately adjacent to the innermost circle indicates the GC-skew [(G − C)/(G + C)]. The direction of the arrow indicates the direction of transcription. The figure was generated using CG view.

**Figure 3:**
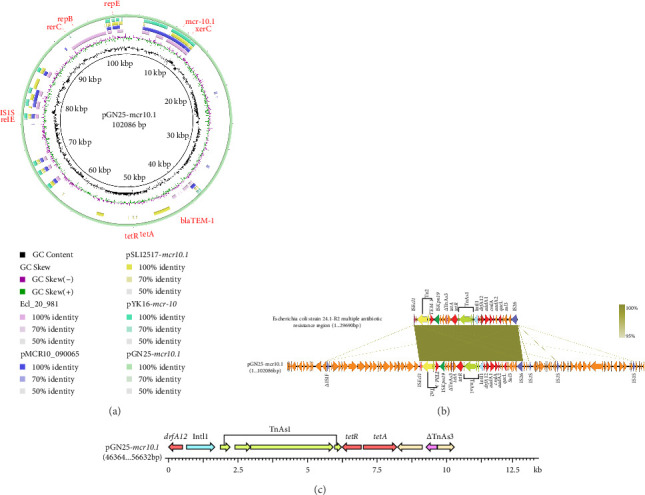
Comparative genomics and genetic architecture of the *mcr-10.1*-harboring plasmid pGN25-*mcr10.1*. (A) comparative analysis of pQDFD216.1 with four closely related *mcr-10.1*-carrying plasmids, including Ecl_20_981 (GenBank accession: CP048651.1), pMCR10_090065 (GenBank accession: CP045065.1), pSL12517-*mcr10.1* (GenBank accession: MW048777.1), and pYK16-*mcr-10* (GenBank accession: MT468575.1). pGN25-*mcr10.1* was used as the reference plasmid for BRIG. (B) linear comparison of gene clusters in the multidrug resistance region of plasmid pGN25-*mcr10.1* and *E. coli* 24.1-R2. The gene backbone is indicated by arrows. Arrows of the same color indicate mobile elements such as various insertion sequences, and red arrows indicate resistance genes. (C) linear schematic of the resistance gene islands carried by plasmid pGN25-*mcr10.1*.

The complete genome of GN25 comprises a circular chromosome with a length of 4,886,792 bp and a GC content of 50.86%, along with a circular plasmid designated pGN25-*mcr10.1* spanning 102,086 bp. Multilocus sequencing typing (MLST) analysis revealed that strain GN25 belonged to sequence type 549 (ST549). The pGN25-*mcr10.1* plasmid was a hybrid of IncFIB and IncX1 harboring the *mcr-10.1* gene. It was found to be a new type of plasmid harboring *mcr-10.1* by comparing the known plasmid types harboring *mcr-10* and was posted to GenBank with the accession number CP139197. Comparative analysis of resistance gene sequences revealed that this plasmid carries the chloramphenicol resistance gene *cmlA*, the *β*-lactam resistance gene *bla*_TEM−1_, the colistin resistance gene *mcr-10.1*, the tetracycline resistance gene *tetA*, the sulfonamide resistance gene *sul3*, the trimethoprim resistance gene *dfrA12*, the quaternary ammonium resistance gene *qacL*, and the aminoglycoside resistance genes *aadA1*, *aadA2*, *APH* (*3′'*)*-Ib*, and *APH* (*6*)*-Id*. These results were consistent with the resistance phenotype, except for *mcr-10*, which did not have a colistin resistance phenotype. The *mcr-10.1* flanking sequences included *xerC* (site-specific tyrosine recombinase), as well as a large number of hypothetical proteins. *xerC*, which mediates the mobilization of genetic elements, as well as the IS elements *Δ*IS*Kox1*, *Δ*IS*903*, and IS*Kpn74* was found both upstream and downstream of this plasmid (Figure 2). Using the current database, Blastn analysis of pGN25-*mcr10.1* showed that this plasmid was somewhat similar to Ecl_20_981 (GenBank accession: CP048651.1), pMCR10_090065 (GenBank accession: CP045065.1), pSL12517-*mcr10.1* (GenBank accession: MW048777.1), and pYK16-mcr-10 (GenBank accession: MT468575.1) (Figure 3A). BLAST comparison of the plasmid sequences revealed a 29,690-bp segment exhibiting 100% identity with the MDR genomic island of *E. coli* (GenBank accession: KX117210.1), and that its MLST was ST549. Specifically, nine antimicrobial resistance genes mediating resistance to *β*-lactams (i.e., *bla*_TEM−1_), chloramphenicol (i.e., *cmlA*), aminoglycosides (i.e., *aadA1* and *aadA2*), quaternary ammonium (i.e., *qacL*), sulfonamide (i.e., *sul3*), trimethoprim (i.e., *dfrA12*), and tetracycline (i.e., *tetA* and *tetR*) were coded by the entire MDR region (Figure 3B). A resistance genomic island was identified at 46,364–56,632 bp of the plasmid and that a class Ⅰ integron was flanked by the diaminopyrimidine resistance genes *dfrA12* and *TnAs1* of the *Tn3* family of transposons, the whole gene island containing the tetracycline resistance genes *tetA* and *tetR* and the trimethoprim resistance gene *dfrA12* (Figure 3C).

### 3.3. Genetic Environment of *mcr-10.1* in *E. coli*

**Figure 4:**
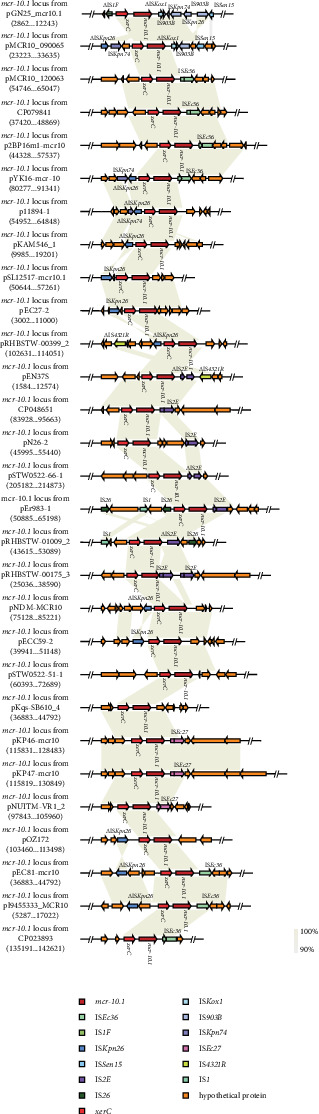
Genetic environment of *mcr-10.1* in *E. coli*. Comparison of 29 *mcr-10.1* loci from 29 plasmids. Genes are indicated by arrows. Genes, insertion sequences, and other features are colored based on their functional classification. Shading denotes regions of homology (nucleotide identity ≥90%). Numbers in brackets indicate nucleotide positions within the 29 plasmids.

Information regarding the plasmids carrying the *mcr-10* gene in the host, source, etc., was collected from GenBank as of November 1, 2023 (Supporting Information 3: Table S2). The plasmids were mainly found in hospital wastewater and were the first *mcr-10* gene carriers to be found in migratory birds. By comparing some of the genes upstream and downstream of the *mcr-10.1* gene carried by the pGN25-*mcr10.1* plasmid, it was found that *xerC* and an *Δ*IS*1F* IS elements were present upstream of the *mcr-10.1* gene, and that IS elements such as *Δ*ISK*ox1*, *Δ*IS*903*, and IS*Kpn74* were present immediately downstream of the *mcr-10.1* gene (Figure 4). As shown in Figure 4, every *mcr-10* gene locus carried *xerC* upstream. Intact or truncated IS elements, transposons, and hypothetical proteins occurred either upstream or downstream of the *xerC-mcr10.1* region. Notably, *xerC* was the only conserved MGE adjacent to *mcr-10.1*, and *mcr-10.1* and was found downstream. Plasmid linear mapping further revealed that plasmids harboring *mcr-10.1* diversify their structures through multiple IS elements or transposons.

### 3.4. Conjugation Experiments

Several conjugation experiments were performed, but none were successful. Comparing the WGS data showed that this plasmid did not have the four conjugative plasmid modules or the integrative and conjugative elements, the oriT region, relaxase, type IV coupling proteins, and bacterial type IV secretion system, showing that this plasmid was non-conjugative [12].

### 3.5. Generation and Antimicrobial Susceptibility Analysis of Plasmid-Cured *mcr-10.1*− Derivative

To investigate the stability of the *mcr-10.1* gene harbored in the plasmid, the GN25 strain was allowed to naturally lose the plasmid through successive passages. After being passed to the 20th generation, the rate of plasmid loss was 8.79% (8/91), and the resulting strain was named GN25-Q (Supporting Information 5: Table S4). The AST results for GN25-Q, obtained using the BD Phoenix−100 Automated Microbiology System, are shown in Supporting Information 4: Table S3. The resistance profile of the GN25-Q strain has undergone a transformation. The resistance profile of the GN25-Q strain has undergone a transformation. In contrast to the GN25 strain, which exhibits resistance to ampicillin, piperacillin, trimethoprim-sulfamethoxazole, chloramphenicol, and tetracycline, the GN25-Q strain has become sensitive to these antibiotics. No significant changes were observed in its resistance to the other antibiotics that were tested. This suggests that the plasmid pGN25-*mcr10.1*, which harbors the resistance genes for these specific antibiotics, was lost, and the resistance genotype now aligns with the resistance phenotype.

### 3.6. Comparative Antimicrobial Susceptibility Analysis of Isogenic Mcr-10.1+/− Variants Following Plasmid-Curing and In Vitro Induction

To discover whether the in vitro application of colistin stress had any effect on the biological properties of *E. coli* harboring the *mcr-10.1* gene, a low concentration of colistin was initially applied to GN25 and GN25-Q *in vitro*, and then gradually increased to apply increasing levels of colistin-resistant stress. The results showed that the parental strains GN25 and GN25-Q could rapidly adapt to colistin stress and successfully induced their daughter strains: *E. coli* GN25-Y, which carried *mcr-10.1* and exhibited a colistin resistance phenotype, and *E. coli* GN25-QY, which did not carry *mcr-10.1* but also exhibited a colistin resistance phenotype. The colistin minimum inhibitory concentration (MIC) values were 16 mg/L for GN25-Y and 32 mg/L for GN25-QY. The ERIC-PCR results for both were consistent with those of the susceptible parental strain GN25. The AST results, obtained using the BD Phoenix−100 Automated Microbiology System, are shown in Table S3. Both strains changed from susceptible to resistant to colistin, and there were no significant changes in their susceptibility to the other antibiotics tested.

### 3.7. Phenotypic Profiling of *E. coli* GN25 and Derived Strains

**Figure 5:**
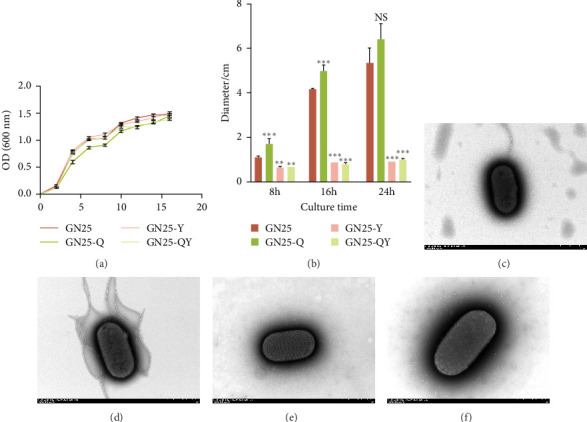
Biological characterization observations. (A) growth curves of GN25, GN25-Q, GN25-Y, and GN25-QY. (B) colony diameters of strains GN25, GN25-Q, GN25-Y, and GN25-QY incubated at 37°C on 03% semi-solid LB agar medium for 8 h, 16 h and 24 h. Symbols indicate differences in the diameter of the bacterial circle compared with GN25 (*⁣*^*∗∗*^*p* < 0.01; *⁣*^*∗∗∗*^*p* < 0.001; NS, no significance). (C) morphology of GN25 observed by transmission electron microscopy at 12,000× magnification. (D) morphology of GN25-Q observed by transmission electron microscopy at 12,000× magnification. (E) morphology of GN25-Y observed by transmission electron microscopy at 12,000× magnification. (F) morphology of GN25-QY observed by transmission electron microscopy at 12,000× magnification.

We assessed the growth kinetics of *E. coli* GN25 and its derivative strains (GN25-Q, GN25-Y, GN25-QY), with growth curves shown in Figure 5A. The growth trend of the parental sensitive strain GN25 was comparable to that of the other derivative strains. There were no significant differences in growth rates among GN25, GN25-Y, and GN25-QY. However, GN25-Q exhibited a slower growth rate compared to the others.

We evaluated motility in *E. coli* GN25 and derivatives using semi-solid agar assays, with the diameter of the migration zone serving as the motility metric (Figure 5B). After 8–16 h incubation, GN25 and GN25-Q formed significantly larger migration zones compared to GN25-Y and GN25-QY (*p* < 0.01). This 81.1%–83.8% reduction in motility among colistin-induced strains (GN25-Y/QY) demonstrates a significant impact of colistin on the motility of *E. coli*.

TEM at 12,000× magnification revealed distinct ultrastructural differences among the *E. coli* GN25 and derivatives strains. Flagellar structures were observed in the colistin-sensitive strains GN25 (Figure 5C) and GN25-Q (Figure 5D), with GN25-Q displaying more flagella compared to GN25. In contrast, no flagella were detected in the colistin-resistant strains GN25-Y (Figure 5E) and GN25-QY (Figure 5F).

## 4. Discussion

Antibiotic resistance presents a growing public health problem globally and the “One Health” concept has been developed to promote regional, national, and global multidisciplinary synergies to achieve optimal conditions for the health of humans, animals, and the environment [13]. However, the role of wild birds in the spread of antibiotic resistance appears to have been underestimated. Their vast flight spans, extensive mobility across diverse habitats, and intricate migratory pathways collectively enable them to disseminate intricate drug-resistant bacterial strains across expansive geographical regions [14]. So far, *mcr-10.1*, which can be carried by *E. cloacae*, *Enterobacter roggenkampii*, *Enterobacter kobei*, *Klebsiella quasipneumoniae*, among others, has been found in China [15], Italy [16], Nepal [17], Korea [18], and Switzerland [19], and elsewhere. Lin et al. isolated highly homologous *E. coli* Incl2 plasmids from human patients in Australia and China and this plasmid was also found in samples from plovers along the East Asia–Australia bird migration route [20].

In this study, the MDR *E. coli* strain GN25 (ST549) isolated from night herons in Nanning, China, shares identical MLST type and a 29,690 bp resistance region with an Australian clinical strain from healthy humans [21]. Given the overlapping migratory routes of Australian night herons through China, we propose avian hosts facilitate intercontinental transmission of ST549 clones across human–animal–environment interfaces. Migratory birds serve as critical vectors for global antibiotic resistance dissemination, acquiring and transporting resistant bacteria along flyways. Of particular concern is the plasmid-borne *mcr-10* gene in colistin-sensitive *E. coli*, enabling stealthy gene transfer to pathogenic hosts.

The *mcr* gene family displays low inactivation rates, with three reported mechanisms: 22-bp repeat disruption [22], IS*1294b* inactivation [23], and IS*10R* inactivation [24]. Genomic analysis of *E. coli* GN25 confirmed that these inactivation events did not occur, yet the strain mostly remained susceptible to colistin (MIC = 1 mg/L). Currently, bacteria reported to carry the *mcr-10.1* gene are sensitive to colistin, but the mechanism underlying the low-level resistance phenomenon mediated by *mcr-10.1* remains unclear [16]. Similar observations have been made for *mcr-9*-carrying *Salmonella* and *Pantoea calida* [25], where susceptibility persisted despite *mcr* presence, potentially due to absent regulatory genes (e.g., qseBC). Contrastingly, *mcr-10.1* combined with *phoPQ* two-component regulatory system can drive high-level resistance (MIC = 8 mg/L) [26]. A previous study demonstrated that *mcr-10.1* was able to cofunction with *phoP* (two-component system response regulator) and *phoQ* (two-component system sensor histidine kinase) to mediate high-level colistin resistance [27]. However, after colistin treatment, both the host bacteria and the plasmid types harboring *mcr-1* may play a regulatory role in the expression of the *mcr-1* gene, suggesting that *mcr-1* may be involved in a complex regulatory network [28]. Notably, GN25 harbored chromosomal *qseB/C* and *phoPQ* genes, yet showed no resistance enhancement, suggesting *mcr-10.1*-mediated low-level resistance may operate independently of these systems.

Structurally, the conserved *xerC-mcr-10.1* locus serves as a genomic anchor, flanked by diverse IS/transposon insertions that drive resistance module plasticity. The regions adjacent to this locus function as MGE integration hotspots, facilitating structural diversification of *mcr-10.1*-bearing plasmids.

Growth curve analysis revealed no measurable growth impairment in *E. coli* strains GN25 and its derivatives. This phenotypic stability is likely attributable to co-evolved compensatory mechanisms between the hybrid plasmid and host chromosomal elements [29, 30]. However, repeated induced passages substantially mitigated these costs, potentially explaining the successful dissemination of such hybrid plasmids [31]. In vitro exposure to colistin significantly suppressed flagellar gene expression in *E. coli* GN25-Y and GN25-QY, concomitant with a decrease in swarming motility compared to untreated controls (*p* < 0.01). Therefore, we hypothesized that the non-expression of colistin resistance genes may facilitate the motility of *E. coli* and improve its adhesion to the host intestine, promoting its symbiotic existence, and paving the way for its evolution towards pan-resistance.

## 5. Conclusion

This is the first report of the identification of the *mcr-10.1* gene isolated from a migratory bird. We discovered that the genetic organization *xerC-mcr-10.1* may be a conserved region of the *mcr-10.1* locus. It could be captured by various MGEs and integrated into diverse types of plasmids. It is particularly important to note that bacteria harboring the *mcr-10.1* gene may exhibit low MIC values, but that the MIC values under colistin selective pressure can become progressively higher and exacerbate the difficulty of treating infections caused by *mcr-10.1*-associated bacteria. Therefore, vigilance for such “silent transmission” is warranted, and continuous monitoring of the spread of *mcr-10.1* is necessary in the future.

## Data Availability

All relevant data are within the paper.

## Supporting Information

Supporting Information 1 The Supporting Information offers supporting explanations pertaining to the methodology section of this article.

Supporting Information 2 Table S1. Source of multidrug-resistant *E. coli* and statistics on resistance genes.

Supporting Information 3 Table S2. General features of the 29 *mcr-10.1*-harbouring plasmids.

Supporting Information 4 Table S3. Results of antimicrobial susceptibility testing, MIC and *mcr-10* PCR assay for 4 strains of *E. coli*.

Supporting Information 5 Table S4. *E. coli* 16S rDNA、*mcr-10* and Eric primer sequences.
