# Supplementary material for: Integrated Genomic and Phenotypic Characterization of an Mcr-10.1-Harboring Multidrug Resistant Escherichia coli Strain From Migratory Birds in China
Source: Transbound Emerg Dis. 2025 May 1;2025:7631217. doi: 10.1155/tbed/7631217 (PMC12061519; doi:10.1155/tbed/7631217)
Supplement: Supporting Information 1 — The Supporting Information offers supporting explanations pertaining to the methodology section of this article. [file 7631217.f1.doc]

**Supplementary Methods**

**1.1 *In vitro* induction of pGN25-mcr10.1 plasmid deletion strain**

Large plasmids often impose substantial metabolic burdens on host bacterial strains, leading to potential plasmid loss during serial passaging in antibiotic-free environments (Ma, Feng, & Zong, 2018). To generate the pGN25-mcr10.1-cured strain, a single colony of *E. coli* GN25 was inoculated into Brain-Heart Infusion (BHI) broth and incubated at 37°C for 12 h. The culture was subsequently subjected to serial passaging by 500-fold dilution in fresh BHI medium followed by 12 h incubation cycles (Li, Du, Zhang, Li, & Bai, 2021). At 10-generation intervals, bacterial suspensions were serially diluted to 10^-5 and plated on BHI agar to isolate single colonies. At 10-generation intervals, bacterial suspensions were serially diluted to 10^-5 and plated on BHI agar to isolate single colonies. Ninety-one randomly selected colonies were screened for plasmid loss through PCR amplification of the *mcr-10* gene. Putative plasmid-cured isolates were further verified by Enterobacterial Repetitive Intergenic Consensus (ERIC)-PCR to exclude cross-contamination. A confirmed plasmid-free strain was designated as GN25-Q.

**1.2 *In vitro* induction of GN25 and GN25-Q resistance**

Both *E. coli* GN25 and its plasmid-cured derivative GN25-Q initially exhibited colistin susceptibility. Resistance phenotypes were induced through stepwise colistin induction as follows: Single colonies were inoculated into BHI broth (37°C, 12 h), followed by serial passaging in media containing incremental colistin concentrations. Primary cultures (200 μL) were transferred at 1:10 dilution into BHI supplemented with 0.25 mg/L colistin. Following 12 h incubation at 37°C with visible turbidity development, cultures were similarly passaged through three consecutive cycles at 0.25 mg/L colistin to stabilize adaptation. Parallel induction series were initiated by doubling the colistin concentration at each passage (0.25 → 0.5 → 1.0 mg/L, etc.) until growth cessation occurred. Colistin-adapted isolates demonstrating growth at maximal achieved concentrations were streaked onto BHI agar. Single colonies were subcultured in BHI broth (37°C, 12 h), with biomass subsequently preserved in 20% (v/v) glycerol saline at -80°C. Genomic DNA was extracted from retained cultures for two verification analyses: (1) ERIC-PCR fingerprinting to confirm clonal relationship with parental GN25, and (2) *mcr-10-*specific PCR to assess gene retention status.

**1.3 Observations on the ultrastructure of *E. coli***

Single colonies of GN25, GN25-Y, GN25-Q, and GN25-QY were inoculated onto BHI agar plates and incubated at 37℃ for 14 h. Four to five single colonies were picked from each group of BHI agar plates and samples taken using the floating method. The samples were adsorbed by placing a copper mesh cover on the bacterial solution for 5 min. The copper mesh was then picked up to absorb the excess liquid and then stained by covering with 20 μL phosphotungstic acid dye for 5 s. The mesh was picked up to absorb the excess liquid, and then dried under incandescent light. The bacterial morphology was observed under a transmission electron microscope.

**1.4 Determination of growth curves**

The growth curves of four bacterial strains, namely GN25, GN25-Q, GN25-Y, and GN25-QY, were determined and compared. Single colonies of each strain were picked and inoculated into 3 mL of LB medium for overnight incubation. A turbidimeter was then used to adjust the turbidity of the bacterial cultures to an optical density (OD) of 0.5 at 600 nm (OD₆₀₀). Subsequently, 10 μL of each bacterial solution was added to 1 mL of LB medium in 16 parallel groups, and the cultures were incubated in a shaker at 37°C and 180 rpm. Every 2 hours, two 1 mL samples were taken from each enriched bacterial culture, and their OD₆₀₀ values were measured using an ultra-micro UV spectrophotometer. The growth curves of GN25, GN25-Q, GN25-Y, and GN25-QY were plotted as line graphs to compare the differences in growth rates among the four strains. The experiment was repeated three times, and the results were averaged.

**1.5 motility evaluation through semi-solid agar cultures**

GN25, GN25-Q, GN25-Y, and GN25-QY were inoculated into 3 mL of LB medium and incubated overnight. Subsequently, a turbidimeter was used to adjust the optical density (OD₆₀₀) of the bacterial cultures to 0.5. Then, 2 μL of each bacterial suspension was placed in the center of a 0.3% LB semi-solid agar medium, and the plates were incubated at 37°C for 24 h. The diameters of the bacterial growth zones were measured at 8, 16, and 24 h and used as evaluation indices to compare the differences in motility among the four strains. The experiment was repeated three times, and the results were averaged.

**REFERENCES**

Li, R., Du, P., Zhang, P., Li, Y., & Bai, L. (2021). Comprehensive Genomic Investigation of Coevolution of mcr genes in Escherichia coli Strains via Nanopore Sequencing. *Glob Chall, 5*(3), 2000014.

Ma, K., Feng, Y., & Zong, Z. (2018). Fitness cost of a mcr-1-carrying IncHI2 plasmid. *PLoS One, 13*(12), e0209706.
