# Supplementary material for: Integrated Genomic and Phenotypic Characterization of an Mcr-10.1-Harboring Multidrug Resistant Escherichia coli Strain From Migratory Birds in China
Source: Transbound Emerg Dis. 2025 May 1;2025:7631217. doi: 10.1155/tbed/7631217 (PMC12061519; doi:10.1155/tbed/7631217)
Supplement: Supporting Information 3 — Table S2. General features of the 29 mcr-10.1-harbouring plasmids. [file 7631217.f3.doc]

**Supplementary Materials**

**Table S2. General features of the 29 *mcr-10.1***-harbouring plasmids

| **Plasmid** | **GenBank accession** | **Total length (bp)** | **Host bacterium** | **isolation source** | **Location** | **Reference** |
| --- | --- | --- | --- | --- | --- | --- |
| pGN25-mcr10.1 | CP139397 | 102086 | *Escherichia coli* | night heron | China | This study |
| pMCR10_090065 | CP045065 | 71775 | *Enterobacter roggenkampii* | NA | China | 1 |
| pMCR10_120063 | CP116250 | 195008 | *Enterobacter roggenkampii* | blood | China | 2 |
| unnamed1 | CP079841 | 156487 | *Escherichia coli* | hospital sewage water | Canada | NA |
| p2BP16m1_mcr10 | CP129516 | 165059 | *Enterobacter asburiae* | water | Czech Republic | NA |
| pYK16-mcr-10 | MT468575 | 117855 | *Enterobacter roggenkampii* | chicken | China | 3 |
| p11894_1 | CP083825 | 131857 | *Enterobacter ludwigii* | throat swab | China | 4 |
| pKAM546_1 | AP026874 | 5702 | *Enterobacter roggenkampii* | NA | Japan | NA |
| pSL12517-mcr10.1 | MW048777 | 58151 | *Enterobacter cloacae* | NA | Sierra Leone | 5 |
| pEC27-2 | CP020091 | 84602 | *Enterobacter cloacae* | urine | Vietnam | 6 |
| pRHBSTW-00399_2 | CP056561 | 137623 | *Enterobacter cloacae* | Wastewater effluent sample | UK | NA |
| pEN37S | AP024497 | 70277 | *Enterobacter cloacae* | Canis lupus familiaris | Japan | NA |
| unnamed1 | CP048651 | 161986 | *Enterobacter roggenkampii* | Medical waste wate | China | NA |
| pN260-2 | AP023449 | 244996 | *Enterobacter roggenkampii* | human bile | Japan | 7 |
| pSTW0522-66-1 | AP022466 | 324199 | *Enterobacter roggenkampii* | hospital sewage | Japan | NA |
| pEr983-1 | CP060738 | 100102 | *Enterobacter roggenkampii* | sewage water | China | 8 |
| pRHBSTW-01009_2 | CP056127 | 70650 | *Enterobacter asburiae* | Wastewater effluent sample | UK | NA |
| pRHBSTW-00175_3 | CP055932 | 68715 | *Enterobacter sp.* | Freshwater sample from upstream of wastewater treatment plant | UK | NA |
| pNDM-MCR10 | NZ_CP135262 | 179424 | *Enterobacter asburiae* | NA | China | NA |
| pECC59-2 | CP080472 | 64293 | *Enterobacter hormaechei* | Broncho-alveolar lavage | China | NA |
| pSTW0522-51-1 | AP022432 | 159829 | *Enterobacter kobei* | hospital sewage | Japan | NA |
| pKqs_SB610_4 | CP084774 | 124980 | *Klebsiella quasipneumoniae* | Water (environment) | Netherlands | NA |
| pKP46-mcr10 | CP088124 | 186056 | *Klebsiella pneumoniae* | chicken | China | NA |
| pKP57-mcr10 | CP088129 | 186040 | *Klebsiella pneumoniae* | chicken | China | NA |
| pNUITM-VR1_2 | AP025011 | 261835 | *Raoultella ornithinolytica* | NA | Vietnam | NA |
| pOZ172 | CP016763 | 127005 | *Citrobacter freundii* | Sun Yat-sen Memorial Hospital | China | 9 |
| pEC81-mcr10 | CP088133 | 62662 | *Escherichia coli* | stool | China | NA |
| pI9455333_MCR10 | CP122443 | 129863 | *Enterobacter ludwigii* | rectal swab | Czech Republic | NA |
| unnamed1 | CP023893 | 231294 | *Raoultella ornithinolytica* | Rectal | Canada | NA |

Note: All the completely sequenced and nonredundant mcr-10.1-carrying plasmids available in GenBank (last accessed 1 November 2023) are included. NA, not applicable.

**REFERENCES**

1. Wang C, Feng Y, Liu L et al. Identification of novel mobile colistin resistance gene mcr-10. *Progress in Artificial Intelligence* 2020; **9**.

2. Wang C, Feng Y, Zong Z. Complete Genome Sequence of an mcr-10-Carrying Enterobacter roggenkampii Strain Isolated from a Human Blood Culture. *Microbiol Resour Announc* 2023.

3. Lei C-W, Zhang Y, Wang Y-T et al. Detection of Mobile Colistin Resistance Gene mcr-10.1 in a Conjugative Plasmid from Enterobacter roggenkampii of Chicken Origin in China. *Antimicrob Agents Chemother* 2020.

4. Liao W, Cui Y, Quan J et al. High prevalence of colistin resistance and mcr-9/10 genes in Enterobacter spp in a tertiary hospital over a decade. *International journal of antimicrobial agents* 2022; **59**: 106573.

5. Guan J, Li L, Zheng L et al. First Report of the Colistin Resistance Gene mcr-10.1 Carried by IncpA1763-KPC Plasmid pSL12517-mcr10.1 in Enterobacter cloacae in Sierra Leone. *Microbiol Spectr* 2022.

6. Le-Ha TD, Le L, Le-Vo HN et al. Characterization of a carbapenem- and colistin-resistant Enterobacter cloacae carrying Tn6901 in blaNDM-1 genomic context. *Infection and Drug Resistance* 2019; **Volume 12**: 733-9.

7. A KU, A HN, B AFA et al. Genomic characterization of clinical Enterobacter roggenkampii co-harbouring bla IMP-1 - and bla GES-5 -encoding IncP6 and mcr-9 -encoding IncHI2 plasmids isolated in Japan. *Journal of Global Antimicrobial Resistance* 2021; **24**: 220-7.

8. TingtingXu, Zhang C, Ji Y et al. Identication of mcr-10 carried by self-transmissible plasmids and chromosome in Enterobacter roggenkampii strains isolated from hospital sewage water. *Environmental Pollution* 2020; **268**.

9. Jianhui, Xiong, Maxime et al. Genome and Plasmid Analysis ofblaIMP-4-Carrying Citrobacter freundii B38. *Antimicrobial Agents & Chemotherapy* 2016.
