# Supplementary material for: Integrated Genomic and Phenotypic Characterization of an Mcr-10.1-Harboring Multidrug Resistant Escherichia coli Strain From Migratory Birds in China
Source: Transbound Emerg Dis. 2025 May 1;2025:7631217. doi: 10.1155/tbed/7631217 (PMC12061519; doi:10.1155/tbed/7631217)
Supplement: Supporting Information 4 — Table S3. Results of antimicrobial susceptibility testing, MIC and mcr-10 PCR assay for 4 strains of E. coli. [file 7631217.f4.doc]

**Supplementary Materials**

**Table S3. Results of antimicrobial susceptibility testing, MIC and *mcr-10* PCR assay for 4 strains of *E. coli***

| Strain name | resistant phenotype | Colistin  MIC（mg/L） | | *mcr-10* |
| --- | --- | --- | --- | --- |
| GN25 | AMP-PIP-SXT-CHL-TET | 1 | Positive, | |
| GN25-Y | AMP-PIP-CST-SXT-CHL-TET | 16 | Positive, | |
| GN25-Q | **/** | 1 | Negative | |
| GN25-QY | CST | 32 | Negative | |

**Note:** AMP, ampicillin; PIP, piperacillin; AMC, CST, colistin; SXT, sulfamethoxazole/trimethoprim; CHL, chloramphenicol; TET, tetracycline; /, No resistant phenotype.
