# Supplementary material for: Integrated Genomic and Phenotypic Characterization of an Mcr-10.1-Harboring Multidrug Resistant Escherichia coli Strain From Migratory Birds in China
Source: Transbound Emerg Dis. 2025 May 1;2025:7631217. doi: 10.1155/tbed/7631217 (PMC12061519; doi:10.1155/tbed/7631217)
Supplement: Supporting Information 5 — Table S4. E. coli 16S rDNA、mcr-10 and Eric primer sequences. [file 7631217.f5.doc]

**Supplementary Materials**

**Table S4. *E. coli* 16S rDNA、*mcr-10*** and Eric primer sequences.

| **Name of primer** | **Primer Sequences（5’-3’）** | **Sequence fragment size** | **Reference** |
| --- | --- | --- | --- |
| ***E. coli* 16S rDNA** | **F：TGTGGGAACGGCGAGTCGGAATAC** | **1467 bp** | 1 |
|  | **R：GGGCGCAGGGGATGAAACTCAAC** |  |  |
| **mcr-10** | **F: AGTCCGTTTGTTCTTGTGGC** | **490 bp** | **This study** |
|  | **R: AGATCCTTGGTCTCGGCTTG** |  |  |
| **Eric** | **F: ATGTAAGCTCCTGGGGATTCAC** | **80~3200bp** | **2** |
|  | **R: AAGTAAGTGACTGGGGTGAGCG** |  |  |

Note: F, forward; R, reverse.

**REFERENCES**

1. Shahi SK, Singh VK, Kumar A. Detection of Escherichia coli and Associated β-Lactamases Genes from Diabetic Foot Ulcers by Multiplex PCR and Molecular Modeling and Docking of SHV-1, TEM-1, and OXA-1 β-Lactamases with Clindamycin and Piperacillin-Tazobactam. *Plos One* 2013; **8**.

2. Versalovic J, Koeuth T, Lupski R. Distribution of repetitive DNA sequences in eubacteria and application to fingerprinting of bacterial genomes. *Nucleic Acids Research* 1991; **19**: 6823-31.
